# High Concentrations of Tilmicosin Promote the Spread of Multidrug Resistance Gene *tol*C in the Pig Gut Microbiome Through Mobile Genetic Elements

**DOI:** 10.3390/ani15010070

**Published:** 2024-12-31

**Authors:** Tao Chen, Minxing Zhao, Majian Chen, Xiaoyue Tang, Yuliang Qian, Xiaoting Li, Yan Wang, Xindi Liao, Yinbao Wu

**Affiliations:** 1 College of Animal Science, South China Agricultural University, Guangzhou 510642, China; chentao5012@163.com (T.C.); ctscaudky@yeah.net (M.Z.); 20222122003@stu.scau.edu.cn (M.C.); little_moon0926@163.com (X.T.); 18229728192@163.com (Y.Q.); ywang@scau.edu.cn (Y.W.); xdliao@scau.edu.cn (X.L.); 2 Phage Research Center, Liaocheng University, Liaocheng 252000, China; lixiaoting@lcu.edu.cn; 3 Maoming Branch, Guangdong Laboratory for Lingnan Modern Agriculture, Maoming 525000, China; 4 National Engineering Research Center for Breeding Swine Industry, College of Animal Science, South China Agricultural University, Guangzhou 510642, China; 5 State Key Laboratory of Swine and Poultry Breeding Industry, College of Animal Science, South China Agricultural University, Guangzhou 510642, China; 6 Guangdong Provincial Key Lab of Agro-Animal Genomics and Molecular Breeding, and Key Lab of Chicken Genetics, Breeding and Reproduction, Ministry of Agriculture and Rural Affair, South China Agricultural University, Guangzhou 510642, China

**Keywords:** tilmicosin, gut microbiome, antibiotic resistance genes, mobile genetic elements, antibiotic excretion

## Abstract

The impact of antibiotic therapy on the spread of antibiotic resistance genes (ARGs) and its relationship to gut microbiota remains unclear. This study investigated changes in ARGs, mobile genetic elements (MGEs), and gut microbial composition following tilmicosin administration in pigs. Thirty pigs were randomly divided into control (CK), low-concentration (0.2 g/kg; L), and high-concentration (0.4 g/kg; H) groups. Tilmicosin concentration in manure peaked on day 16 of dosing and dropped below detectable levels by day 13 of the withdrawal period. While tilmicosin did not significantly affect the total abundance of macrolide resistance genes (MRGs) (*p* > 0.05), it significantly increased the abundance of the multidrug resistance gene *tol*C in the H group compared with the L and CK groups during the withdrawal period (*p* < 0.05). This increase was associated with a coincidental rise in the abundance of MGEs (e.g., *int*1 and *int*2) and the growth of potential *tol*C-hosting bacteria such as *Paenalcaligenes* and *Proteiniclasticum*. Redundancy analysis showed gut microbial composition as the primary driver of MRG abundance, with MGEs, tilmicosin concentration, and manure physicochemical properties playing secondary roles. These findings suggest that high-dose tilmicosin may alter the gut microbiota and promote ARG spread via MGE-mediated transfer.

## Simple Summary

Antibiotic resistance is a growing concern that affects both human and animal health, making infections harder to treat. This study focused on how the antibiotic tilmicosin affects the spread of antibiotic resistance genes in the gut microbiome of pigs. The pigs were given different concentrations of tilmicosin, and their manure was analyzed to track changes. The results showed that while there was no significant change in the total abundance of macrolide resistance genes, the high-concentration treatment led to a significant increase in the abundance of the multidrug resistance gene *tol*C. This gene has been associated with changes in the gut microbiota, particularly an increase in certain host bacteria. This study is important because it highlights how antibiotics can impact the gut microbiome and promote the transfer of resistance genes, which could ultimately affect both animal and human health.

## 1. Introduction

The increasing prevalence of antibiotic resistance has emerged as a significant public health concern worldwide, with serious implications for the effectiveness of medical treatments. Antibiotics are routinely administered in livestock, not only for therapeutic purposes but also for growth promotion and disease prevention [1,2]. While many countries have banned the use of antibiotics as growth promoters, therapeutic antibiotics continue to be widely used in livestock [3], raising concerns about their impact on the gut microbiota of pigs and the potential for the spread of antibiotic resistance genes (ARGs) from livestock to humans in these areas.

The intestinal microbiota of pigs plays a crucial role in their overall health and well-being, contributing to digestion, nutrient absorption, and immune function. However, antibiotic treatment can disrupt the delicate balance of this microbial community, leading to dysbiosis, which is characterized by a decrease in microbial diversity and the proliferation of pathogenic bacteria [4]. This disruption can facilitate the spread of ARGs through mechanisms such as horizontal gene transfer (HGT). The Global Antimicrobial Resistance Monitoring Report highlights a direct correlation between increased antibiotic usage and bacterial resistance [5]. Therefore, antibiotic treatment not only impacts the gut microbiota, but may also promote the spread of antibiotic resistance genes by inducing dysbiosis, posing a potential risk to human health.

Macrolide antibiotics, including tilmicosin, are pivotal in clinical livestock treatment, and are widely employed to combat infectious diseases in pigs, chickens, and other livestock species [6]. Known for their extended half-life and potent antibacterial properties, macrolides such as tilmicosin effectively target a broad spectrum of pathogens, including Gram-positive and some Gram-negative bacteria, mycoplasmas, and spirochetes [1]. Livestock exhibit weak absorption and metabolism of antibiotics, and the vast majority of antibiotics are excreted through the digestive system. Studies have shown that antibiotics have a selective effect on ARG host bacteria that can lead to the accumulation of ARGs [7,8]. Theoretically, the use of antibiotics in livestock may lead to the enrichment of ARGs. For example, the abundance and diversity of ARGs increased after antibiotic treatment [9]. However, in practice, some antibiotics may also not significantly increase the abundance of ARGs, which may be related to the type of antibiotic, the duration of treatment, or the concentration of the drug administered [10]. Although there are no studies on the effects of tilmicosin on ARGs in pig gut, several studies have shown a generally high detection rate for macrolide resistance genes (MRGs) in pig manure [11,12].

Moreover, the environmental persistence of ARGs poses additional risks. Studies have detected the presence of ARGs in agricultural environments, such as soil and water [13], particularly in areas where manure from treated livestock is applied. This environmental reservoir of resistance genes can pose significant public health risks [14], as resistant bacteria can be transmitted to humans through various pathways, including the consumption of contaminated food and water, occupational exposure, and direct contact with livestock [15,16]. Pathogenic bacteria can acquire ARGs through HGT, complicating infection treatment and increasing healthcare costs, thereby highlighting the need for enhanced surveillance and risk assessment strategies.

In light of these concerns, this study aimed to investigate how high concentrations of tilmicosin affect the intestinal microbiota in pigs and the implications for the spread of ARGs. By examining the changes in the abundance of ARGs, MGEs, and bacterial flora in pig manure following tilmicosin treatment, we aimed to elucidate the potential risks associated with antibiotic use in livestock and the implications for human exposure to resistant pathogens. Understanding these dynamics is crucial for developing effective strategies to mitigate the risks of antibiotic resistance transmission from livestock to humans and to safeguard public health.

## 2. Materials and Methods

### 2.1. Sampling Sites and Sample Collection

Duroc–Landrace–Yorkshire barrows, averaging 32.10 ± 3.15 kg (*n* = 30), were selected from genetically similar populations as experimental subjects. The experimental pigs were randomly divided into three groups: CK (control), L (low dose), and H (high dose). All groups received diets tailored to meet their nutrient requirements, as previously specified [17]. The diets for groups CK, L, and H contained 0 g/kg, 0.2 g/kg, and 0.4 g/kg of tilmicosin, respectively. The dosage of tilmicosin used in this study adhered to the guidelines set forth in the “*Veterinary Pharmacopoeia of the People’s Republic of China (2015)*”.

The pigs underwent a 7-day adaptation period to acclimate to the experimental environment. The total experimental period lasted 29 days, with the medication administered from days 1 to 15 and a subsequent drug withdrawal period from days 16 to 29. Each group randomly selected 6 pigs, from whom fresh pig manure was collected daily. Fresh swine fecal samples can effectively assess the dynamics of gut microbiota.

### 2.2. DNA Extraction and Bacterial 16S rRNA Gene Sequencing

Pig manure samples from days 0, 1, 6, 16, 18, 22, and 29 were used for DNA extraction. Microbial DNA was extracted using an Omega E.Z.N.A. TM Soil DNA Kit (Omega Bio-tek, Norcross, GA, USA), following the manufacturer’s instructions. The process of sequencing library construction, quality assessment, sequencing, and data analysis is detailed in Text S1.

### 2.3. Quantification of Antibiotic Resistance Genes

In this study, 7 MRG subtypes (*erm*A, *erm*B, *erm*C, *erm*F, *erm*Q, *erm*X, and *mef*A), 1 multidrug resistance gene (*tol*C), 4 mobile genetic elements (MGEs) (*int*1, *int*2, *tn*916, and *tnp*A), and the 16S rRNA genes were quantitatively analyzed (Table S1). All qPCR experiments were conducted using a Bio-Rad CFX96 PCR System (Bio-Rad, Hercules, CA, USA).

Recombinant plasmids containing antibiotic resistance genes (ARGs) were constructed as previously outlined [18]. Standard curves were generated by subjecting each plasmid to 10-fold serial dilution, and these diluted plasmids were then utilized as templates for qPCR. Both absolute and relative gene abundances were calculated as described in previous studies [19].

### 2.4. Measurement of Physicochemical Properties

The manure pH was measured in a 1:5 manure-to-water suspension using a pH meter.

The moisture content (MC) of the manure was determined after drying in an oven (Hengfeng, Hanchuan, China) at 105 °C. The electrical conductivity (EC) of the manure was measured using an EC meter following the mixing of 2 g of manure with 20 mL of water (INESA, Shanghai, China). Levels of total nitrogen (TN), organic matter (OM), ammonium nitrogen (NH_4_^+^-N), and nitrate nitrogen (NO_3_^−^-N) in the manure were analyzed using methods previously described [20].

### 2.5. Detection of Tilmicosin in Pig Manure

Tilmicosin concentrations in pig manure were measured using LC–MS (Agilent 1200-Agilent G6410B, Santa Clara, CA, USA), with sample pretreatment and detection methods detailed in Text S2.

### 2.6. Data Analysis

Differences between groups in ARG and MGE abundance and physicochemical properties were analyzed using SPSS 20.0 (IBM, Armonk, NY, USA). STAMP 2.1.3 was used to analyze differences in bacterial community composition in pig manure. Network diagrams, based on Pearson’s correlation coefficients (R > 0.7, *p* < 0.05), were generated using Cytoscape 3.8.0 (Cytoscape Consortium, San Diego, CA, USA). Redundancy analysis (RDA) was performed using R 4.1.1.

## 3. Results and Discussion

### 3.1. Residual Concentrations in Pig Manure After Tilmicosin Treatment

**Figure 1:**
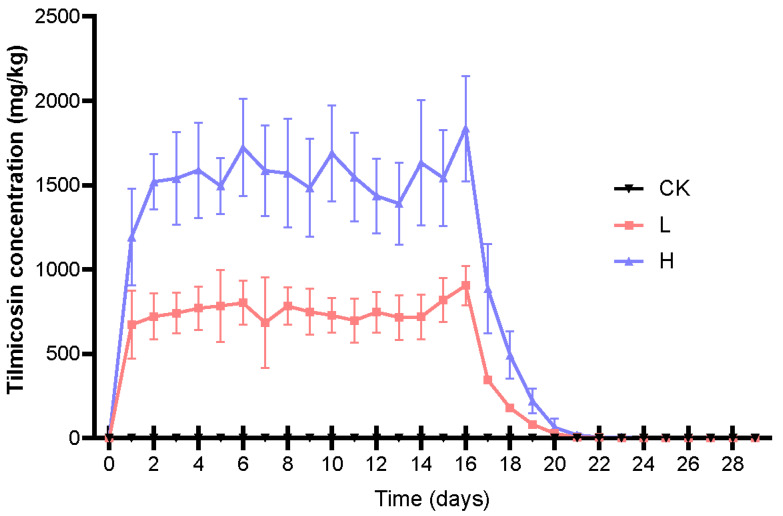
Changes in the concentration of tilmicosin in pig manure from each experimental group (n = 6). Data are expressed as mean ± SD based on dry matter (DM).

The study results indicated that the administration of tilmicosin did not significantly impact the daily feed intake or manure production in the pigs (*p* > 0.05) (Figure S1). On the first day of treatment, tilmicosin concentrations in fresh pig manure rose sharply (Figure 1), suggesting a high rate of absorption and wide distribution within the animals. Previous studies have reported that tilmicosin is rapidly absorbed, with peak blood concentrations occurring approximately 3 h post-administration [21]. The mean concentrations of tilmicosin reached their highest levels on day 16, measuring 911.1 ± 228.6 mg/kg and 1858.5 ± 438.1 mg/kg in groups L and H, respectively.

After treatment, tilmicosin concentrations decreased rapidly, falling below detection limits by day 13 of the withdrawal period, indicating a notable residual time of up to 13 days in pigs. Moreover, the total tilmicosin excretion rate was significantly higher in group H than in group L (*p* < 0.05) (Table S4). Notably, in general, tilmicosin was highly persistent in manure, with a DT50 > 200 days [22]. Studies have shown that the residual times of tilmicosin in pig manure and soil range from 38 to 346 days and 16 days [23], respectively.

This long residual time of tilmicosin in pig manure can have environmental toxicological effects and can have a selective effect on resistant bacteria, promoting the spread of ARGs [24]. Considering the environmental implications of such residuals, the use of fresh pig manure samples to study the dynamics of antibiotic residues is valuable for accurately monitoring and mitigating their ecological impact, especially with respect to ARG dissemination.

### 3.2. Abundance of ARGs in Pig Gut Microbiome After Tilmicosin Treatment

**Figure 2:**
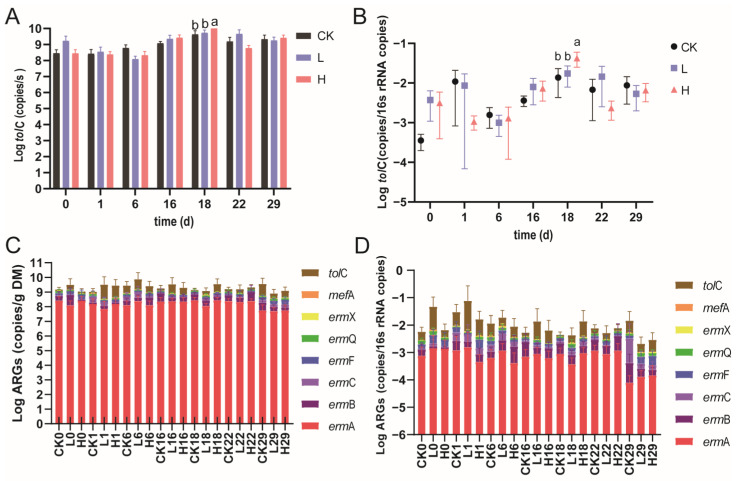
Abundance of ARGs in pig manure after tilmicosin treatment. Absolute (**A**) and relative (**B**) abundance of *tol*C, total absolute abundance (**C**), and total relative abundance (**D**) of ARGs. Groups labeled with different letters indicate significant differences (*p* < 0.05).

In this study, the detection rates for MRGs (*erm*A, *erm*B, *erm*C, *erm*F, *erm*Q, *erm*X, and *mef*A) and the multidrug resistance gene *tol*C reached 97% in pig manure (Tables S5 and S6). On day 18, both the absolute and relative abundances of the multidrug resistance gene *tol*C were significantly higher in group H compared with group CK (*p* < 0.05) (Figure 2A,B), indicating that exposure to high concentrations of tilmicosin enhanced the persistence and spread of *tol*C in the pig gut microbiome. The efflux pump AcrAB-TolC, associated with *tol*C, has diverse substrates and is capable of effluxing various antibiotics, including tilmicosin, from the intracellular compartment [25,26]. In addition to antibiotic resistance, AcrAB-TolC has been associated with other cellular processes, including pathogenicity in infections, bacterial biofilm formation, and adaptation to environmental stress [27,28].

Despite the significant increase in *tol*C abundance, the total abundance (absolute and relative) of MRGs in fresh pig manure did not show significant differences among the treatment groups (*p* > 0.05; Figure 2C,D). A previous study also showed that feeding with telomycin and virginiamycin did not significantly affect MRG abundance in pigs [29]. Compared with the CK treatment, the low-concentration tilmicosin treatment did not significantly affect the abundance of MRG subtypes (*p* > 0.05), and the high-concentration tilmicosin treatment significantly affected the abundance of only *erm*A (increased in absolute abundance on day 6) and *erm*X (decreased in relative abundance on day 6) (*p* < 0.05) (Figures S2 and S3). Another study reached a similar conclusion, showing that the absolute and relative abundances of *erm*A, *erm*B, *erm*C, and *erm*F in cow manure did not change significantly during 21 weeks of feeding with a low concentration of tylosin (11 mg/kg of feed, DM) [30]. Notably, although tilmicosin treatment at high concentrations affected the abundance of *erm*A and *erm*X in manure, this effect was transient.

Furthermore, tilmicosin’s long half-life contributes to its persistence in the pig’s gut and fecal matter, potentially exerting a prolonged selective effect on drug-resistant bacteria. This persistence can extend the selective pressure on ARGs like *tol*C even after active antibiotic treatment [31,32]. Thus, while *tol*C enrichment in the gut microbiome is a primary concern, the lasting presence of tilmicosin in manure underscores an additional environmental risk that merits attention.

### 3.3. Cooccurrence of ARGs and MGEs

**Figure 3:**
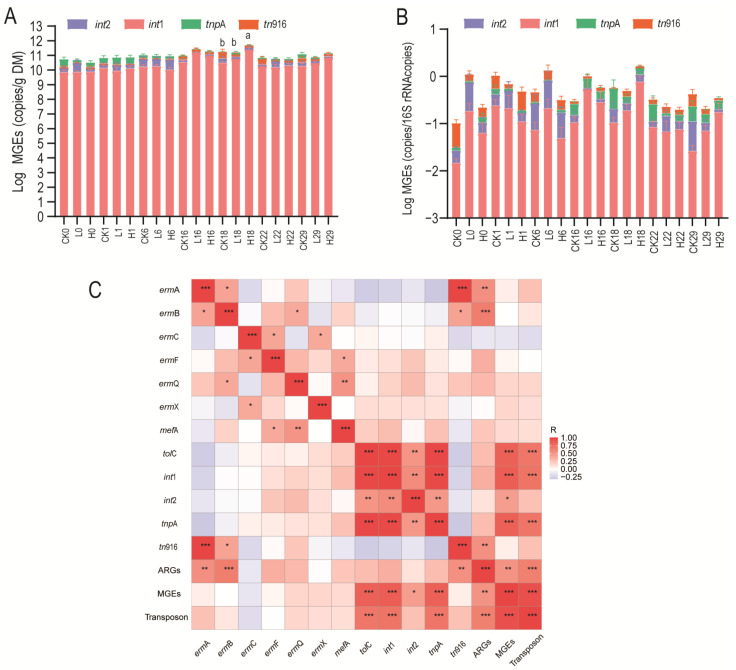
Abundance of MGEs in pig manure after tilmicosin treatment. Absolute (**A**) and relative (**B**) abundance of MGEs and (**C**) Pearson’s correlation coefficient of ARGs and MGEs. * 0.01 < *p* < 0.05, ** *p* < 0.01, *** *p* < 0.001. Groups labeled with different letters indicate significant differences (*p* < 0.05).

HGT is the primary mechanism through which bacteria acquire ARGs, facilitated by mobile genetic elements (MGEs). In this study, the detection rates for MGEs (*int*1, *int*2, *tnp*A, and *tn*916) reached 97% in pig manure (Tables S5 and S6). The total absolute abundance of MGEs throughout the trial period showed a trend of first increasing and then decreasing. The absolute abundance of MGEs in group H was significantly greater than that in groups CK and L on day 18 (*p* < 0.05), but there was no significant difference in the relative abundance of MGEs at the same time point (*p* > 0.05) (Figure 3A,B). Similarly, the absolute abundances of *int*1 and *int*2 in group H were significantly greater than those in group CK and group L (*p* < 0.05) (Figures S4 and S5). Consistent with the *int*1 and *int*2 abundance results, the abundance of *tol*C in group H also significantly increased (*p* < 0.05) (Figure 2A,B), so there may be a potential association between them.

Pearson’s correlation was employed to examine the relationships among the abundances of eight ARGs and four MGEs (Figure 3C). The results showed that MRGs were significantly positively correlated with the MGEs (*p* < 0.05). The *tol*C abundance was significantly positively correlated with *int*1, *int*2, and *tnp*A (*p* < 0.05). Previous studies have demonstrated that *int*1 and *tnp*A play significant roles in HGT [33,34], and *int*1 is the most widely distributed and abundant MGE in the environment [35]. The above results indicated that tilmicosin treatment may lead to a significant increase in the abundance of *int*1 and *int*2 in pig manure, which in turn leads to a significant increase in *tol*C abundance through HGT.

### 3.4. Effects of Tilmicosin Treatment on the Bacterial Flora in Pig Gut Microbiome

**Figure 4:**
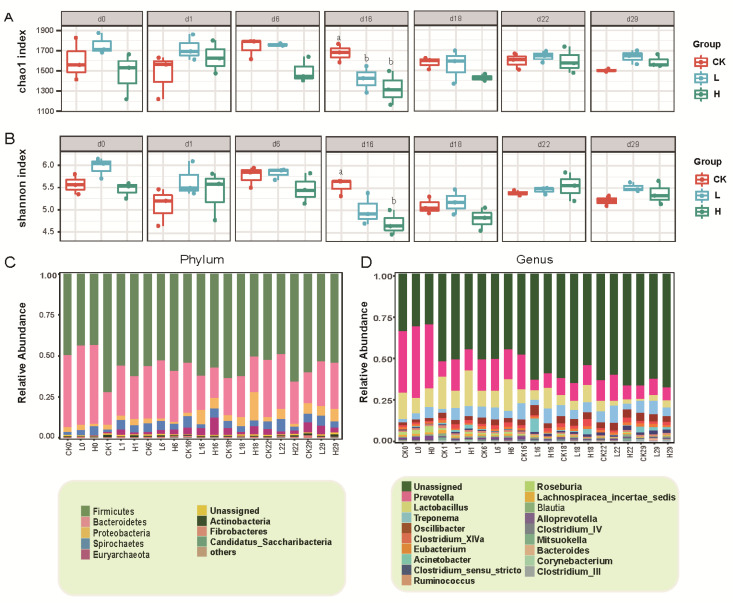
Changes in the diversity and structure of the pig manure flora following tilmicosin treatment. (**A**,**B**) α diversity assessed by Chao1 and Shannon indices, (**C**) relative abundance of bacteria at the phylum level, and (**D**) relative abundance of bacteria at the genus level. Groups labeled with different letters indicate significant differences (*p* < 0.05).

In this study, the Chao1 and Shannon indices tended to first decrease and then increase in both groups H and L (Figure 4A,B). Tilmicosin treatment triggered a pattern of a decrease followed by an increase in the Chao1 and Shannon indices. Moreover, tilmicosin treatment significantly decreased the α diversity of the bacterial flora on day 16 (*p* < 0.05) (Figure 4A,B). PCoA was employed to assess the β diversity of the flora. On day 0, the CK, H, and L groups clustered closely together. Following tilmicosin treatment, the H group gradually diverged from the CK and L groups. On day 18, the H group was completely separated from the L and CK groups (Figure S6). Bacterial diversity contributes to the stabilization of gut bacteria, whereas antibiotic exposure leads to a decrease in the diversity of bacterial flora, which may adversely affect pig health [36,37,38].

Antibiotic exposure not only diminishes bacterial flora diversity but also markedly impacts bacterial community structure [36]; therefore, this study also examined the effect of tilmicosin treatment on the composition of the bacterial flora. The dominant phyla consisted mainly of Firmicutes, Bacteroidetes, Proteobacteria, and Actinobacteria. The dominant genus was *Prevotella*, which had an average abundance of 14.2% in all the samples (Figure 4C,D). The abundance of genera such as *Roseburia* changed significantly on days 16 and 18 due to the use of high concentrations of tilmicosin (*p* < 0.05) (Figure S7). The bacterial flora was stable after antibiotic exposure and could be recovered within a certain period [36,39]. There was no significant difference in the abundance of bacterial genera among the CK, H, and L groups on day 22, suggesting that the structure of the bacterial flora was restored.

In summary, tilmicosin administration resulted in significant yet reversible effects on the diversity and composition of bacterial flora, as reflected in the pig manure.

### 3.5. Effects of Tilmicosin Treatment on Potential Host Bacteria for ARGs in the Pig Gut Microbiome

**Figure 5:**
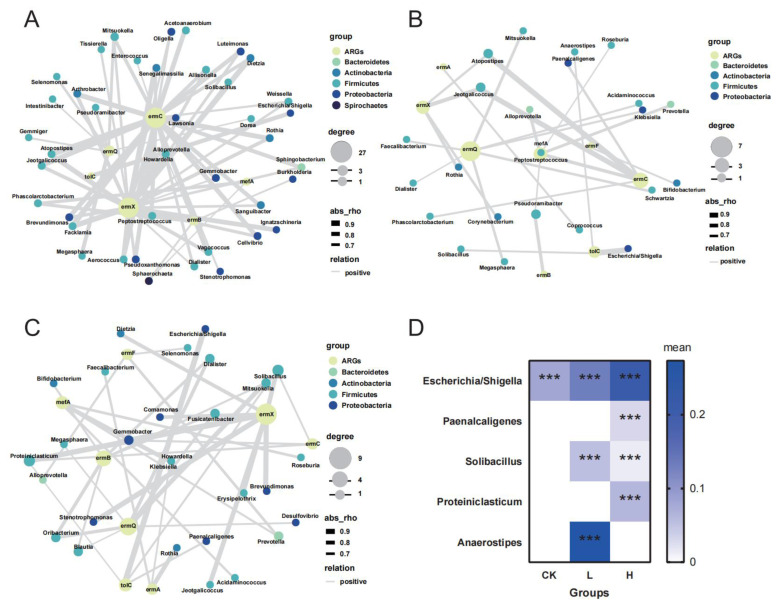
Potential host bacteria for ARGs. (**A**) Network analysis of the CK group, (**B**) network analysis of the L group, (**C**) network analysis of the H group, and (**D**) mean abundance of *Escherichia/Shigella*, *Paenalcaligenes*, *Solibacillus, Proteiniclasticum*, and *Anaerostipes* in the CK, L, and H groups and their correlation with *tol*C. *** *p* < 0.001.

The composition of the gut bacterial flora is a primary factor influencing the distribution and proliferation of ARGs [13,40]. Bacteria exhibiting strong positive associations with ARGs are frequently identified as potential hosts [41,42]. In this study, we used a network analysis approach to explore the relationship between ARGs and their potential host bacteria [43]. Following tilmicosin exposure, notable differences emerged in the potential host bacteria for ARGs within the pig gut microbiome. Specifically, we found 68, 26, and 39 potential host bacteria in the CK, L, and H groups, respectively (Figure 5). As reported in previous studies, most ARG-host bacteria identified belonged to the Firmicutes phylum, suggesting a key role for Firmicutes in ARG dissemination in response to tilmicosin treatment [44].

These findings suggest that tilmicosin exposure may have led to a reduction in the diversity of MRG-host bacteria in both the L and H groups compared with the CK group, particularly due to a decrease in Firmicute and Proteobacteria populations, along with the complete elimination of Spirochaetes. This reduction may be associated with to tilmicosin’s effect on limiting the HGT of resistance genes among genera, thus narrowing the potential ARG host range [45]. Notably, in the CK group, *Escherichia*/*Shigella* were the primary potential hosts for the multidrug resistance gene *tol*C (*p* < 0.05), whereas in the H group, additional genera, including *Solibacillus*, *Paenalcaligenes*, and *Proteiniclasticum,* were also identified as *tol*C hosts (*p* < 0.05) (Figure 5D). The increased abundance of *tol*C in group H may have been due to the enhanced diversity and prevalence of potential ARG-host bacteria in response to high-dose tilmicosin treatment (Figure 2A,B). These findings highlight the need for close monitoring of multidrug resistance gene shifts within the pig gut microbiome following therapeutic antibiotic exposure, as these shifts could impact ARG spread through manure management practices [46].

### 3.6. Effects of Antibiotics, Physicochemical Properties, Bacterial Flora, and MGEs on ARGs

We characterized the effect of tilmicosin treatment on the physicochemical properties of pig manure. The results showed that the MC, OM, and NH_4_^+^-N did not change significantly (*p* > 0.05), but the pH, EC, NO_3_^−^-N, and TN changed significantly (*p* < 0.05) (Figure S8). Antibiotic use can impact the metabolic activity of the intestinal flora, subsequently altering the amount of TN and NO_3_^−^-N in manure [36], which is consistent with our research findings.

**Figure 6:**
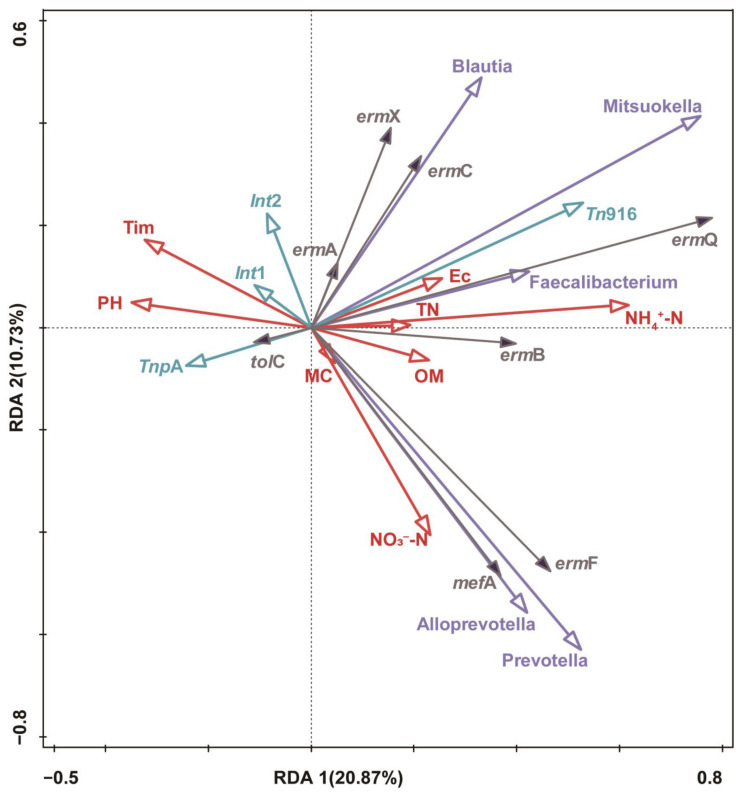
Impacts of antibiotics, bacterial flora, physicochemical properties, and MGEs on ARGs. Black arrows, red arrows, and bluish-purple arrows represent ARGs, physicochemical properties, and bacteria, respectively.

Environmental factors can influence bacterial flora in complex environments, thereby impacting the distribution of ARGs [13,40]. Hence, this study investigated the impact of physicochemical properties, bacterial flora, and MGEs on ARG distribution using RDA. Previous studies have shown that reducing the abundance of ARGs is primarily accomplished by limiting the diversity of their bacterial hosts [40]. In this study, MRGs such as *erm*B, *erm*F, and *erm*Q in pig manure showed strong positive correlations with the dominant bacterial genera *Prevotella*, *Alloprevotella*, *Blautia*, *Mitsuokella*, and *Faecalibacterium*, but all the MRGs were negatively correlated with tilmicosin concentration, which is consistent with the finding that the effect of tilmicosin trans-feeding on the abundance of MRGs in pig manure was not significant. Notably, *tol*C was significantly and positively correlated with the abundance of *int*1, *int*2, and *tnp*A, and the concentration of tilmicosin (*p* < 0.05), suggesting that a significant increase in the absolute abundance of *int*1 and *int*2 led to a significant increase in the abundance of *tol*C (Figure 6). Both bacterial flora and macrolides are drivers of ARGs in the environment [47]. The present study showed that the bacterial flora was the most important factor influencing MRGs in pig manure. In general, the dominant bacterial genera *Prevotella, Alloprevotella*, *Blautia*, *Mitsuokella*, and *Faecalibacterium* play important roles in the absolute abundance of MRGs, followed by MGEs, tilmicosin, and the physicochemical properties of pig manure.

### 3.7. Limitations of the Current Study and Recommendations for Future Research

This study provides valuable insights into the effects of tilmicosin on ARG abundance and distribution within the pig gut microbiome, using fresh fecal samples as proxies. However, there are several limitations that should be acknowledged. Although fecal samples are a practical and non-invasive approach for monitoring changes in the gut microbiota, they may not fully capture the diversity and metabolic complexity of the entire gut microbial ecosystem, particularly the bacteria that adhere closely to the gut wall. Moreover, microbial compositions can shift rapidly after excretion, which could potentially affect the temporal accuracy of fecal-based assessments. Additionally, the short duration of the study limited our understanding of the long-term persistence and transmission dynamics of ARGs, especially in different environmental contexts.

Future research should aim to integrate direct intestinal sampling or combine fecal data with analyses of gut wall-associated microbiota to provide a more comprehensive view of microbial changes. Extending the duration of monitoring and exploring the combined effects of multiple antibiotics on ARG dynamics over time could also help deepen our understanding of their long-term impacts.

## 4. Conclusions

In this study, the therapeutic dose of tilmicosin did not significantly affect the abundance of MRGs (*p* > 0.05). However, this study revealed that high concentrations of tilmicosin significantly increased the absolute abundance of the multidrug resistance gene *tol*C and the MGEs *int*1 and *int*2 in pig manure (*p* < 0.05). The multidrug resistance gene *tol*C may undergo HGT in host bacteria such as *Paenalcaligenes* and *Proteiniclasticum* through *int*1 and *int*2. Additionally, RDA revealed that the bacterial flora was the most important factor influencing the abundance of MRGs in pig manure, followed by MGEs, tilmicosin concentration, and the physicochemical properties of pig manure. Our study provides a theoretical basis for an accurate assessment of the ecotoxicological effects of veterinary antibiotics and for preventing environmental contamination and the spread of ARGs from livestock.

## Data Availability

The raw sequence data reported in this paper have been deposited in the Genome Sequence Archive BIG Data Center, Beijing Institute of Genomics (BIG), and the Chinese Academy of Sciences under accession number CRA019891, publicly accessible at https://bigd.big.ac.cn/gsa/ (accessed on 23 October 2024).

## Supplementary Materials

The following supporting information can be downloaded at: https://www.mdpi.com/article/10.3390/ani15010070/s1, Text S1: 16S rRNA gene sequencing analysis workflow; Text S2: Detection of antibiotic residues; Table S1: Primers and PCR conditions for target genes; Table S2. Mobile phase gradient elution procedure; Table S3: Mass spectrum conditions for tilmicosin; Table S4: Excretion rate of tilmicosin in the feces of pigs in each test group; Table S5: Log values of ARG and MGE absolute abundance in pig manure from different days of treat groups (copies/g); Table S6: Log values of ARG and MGE relative abundance in pig manure from different days and treatment groups (copies/g); Figure S1: Effect of tilmicosin on daily feed intake (A) and daily manure production in pigs (B); Figure S2: Absolute abundance of MRGs in pig manure from different days and treatment groups; Figure S3: Relative abundance of MRGs in pig manure from different days and treatment groups; Figure S4: Absolute abundance of MGEs in pig manure from different days and treatment groups; Figure S5. Relative abundance of MGEs in pig manure from different days and treatment groups; Figure S6: Analysis of β-diversity of pig manure in each experimental group; Figure S7: The difference between the three groups at the phylum and genus level; Figure S8: Changes in the main physicochemical properties of pig manure during the experiment. References [48,49,50,51,52] are cited in the supplementary materials.
