# Supplementary material for: High Concentrations of Tilmicosin Promote the Spread of Multidrug Resistance Gene tolC in the Pig Gut Microbiome Through Mobile Genetic Elements
Source: Animals (Basel). 2024 Dec 31;15(1):70. doi: 10.3390/ani15010070 (PMC11718906; doi:10.3390/ani15010070)
Supplement: Supplementary file 1 [file animals-15-00070-s001.zip › animals-3374738-supplementary.pdf]

High Concentrations of Tilmicosin Promote the Spread of Multidrug Resistance Gene *tolC* in the Pig Gut Microbiome Through Mobile Genetic Elements

Tao Chen [1], Minxing Zhao [1], Majian Chen [1], Xiaoyue Tang [1], Yuliang Qian [1], Xiaoting Li [2], Yan Wang [1,3,4,5,6], Xindi Liao [1,3,4,5,6] and Yinbao Wu [1,3,4,5,6,*]

[1] College of Animal Science, South China Agricultural University, Guangzhou 510642, China; chentao5012@163.com (T.C.); ctscaudky@yeah.net (M.Z.); 20222122003@stu.scau.edu.cn (M.C.); little_moon0926@163.com (X.T.); 18229728192@163.com (Y.Q.); ywang@scau.edu.cn (Y.W.); xdliao@scau.edu.cn (X.L.)
[2] Phage Research Center, Liaocheng University, Liaocheng 252000, China; lixiaoting@lcu.edu.cn
[3] Maoming Branch, Guangdong Laboratory for Lingnan Modern Agriculture, Maoming 525000, China
[4] National Engineering Research Center for Breeding Swine Industry, College of Animal Science, South China Agricultural University, Guangzhou 510642, China
[5] State Key Laboratory of Swine and Poultry Breeding Industry, College of Animal Science, South China Agricultural University, Guangzhou 510642, China
[6] Guangdong Provincial Key Lab of Agro-Animal Genomics and Molecular Breeding, and Key Lab of Chicken Genetics, Breeding and Reproduction, Ministry of Agriculture and Rural Affair,
South China Agricultural University, Guangzhou 510642, China
* Correspondence: wuyinbao@scau.edu.cn

Supplementary materials

Text S1 16S rRNA gene sequencing analysis workflow

The hypervariable regions (V3 to V4) of the bacterial 16S rRNA gene were amplified using the barcoded bacterium-specific primers 341 F (5′-CCTAYGGGRBGCASCAG-3′) and 806 R (5′-GGACTACHVGGGTWTCTAAT-3′). After the reaction, mixed PCR products were purified with a GeneJET™ Gel Extraction Kit (Thermo Scientific). Sequencing libraries were then generated using an Ion Plus Fragment Library Kit 48 (Thermo Scientific) following the manufacturer's recommendations. Library quality was assessed on a Qubit 2.0 fluorometer (Thermo Scientific). Finally, the library was sequenced on a NovaSeq 6000 platform (Novogene, China). The 16S rRNA gene sequences were processed using USEARCH 10 (Edgar, 2013) and VSEARCH 2.15 (Rognes et al., 2016). Sequences were clustered into operational taxonomic units (OTUs) at a 97% similarity threshold. Taxonomy was assigned to each OTU using the RDP classifier 80% confidence threshold trained on the SILVA database (Wang et al., 2007). The OTUs assigned to archaea were removed. The Shannon and Chao1 indices were used to measure bacterial α diversity. Bacterial β diversity was measured by the Bray–Curtis index and visualized by principal coordinate analysis (PCoA) [48-51].

Text S2 Detection of antibiotic residues

Approximately 1.0 g of pig manure sample was sonicated with 10 mL of 1%

ammoniated acetonitrile in a 50 mL centrifuge tube for 20 min, followed by vortexing for 5 min and centrifugation at 8000 rpm for 5 min. Take the supernatant into another 50 mL centrifuge tube; Add 10 mL 1% ammoniated acetonitrile to the bottom residue and repeat extraction once. The supernatant was mixed for 1 min with two times of supernatant, and then mixed with vortex for 1 min. Then 3.0 mL of supernatant was measured in another 10 mL centrifuge tube, nitrogen was blown to nearly dry at 40℃, and 3 ml acetonitrile was added for dissolution. Vortex for 1 min, ultrasonic for 1 min. The eluent was filtered through a 0.22 μm membrane for LC-MS detection [52].

LC-MS determination conditions: Chromatographic conditions: Athena C18-WP, 100A column (2.1×150 mm, 5 um); Column temperature 30 ℃, sample room temperature 20 ℃; The sample volume was 10 ul, the flow rate was 0.3 ml/min, and the mobile phase was 0.1% formic acid water and acetonitrile solution. The mobile phase gradient elution procedure is shown in Table S2.

Mass spectrum conditions: In the multi-reaction monitoring mode (MRM), an electrospray ion source (ESI, positive ion mode) was used, the capillary voltage was 4000V, the drying temperature was 300℃, the drying gas flow rate was 6L/min, and the atomizer pressure was 15psi. The mass spectrum conditions of timicosin are shown in Table S3.

Under the above conditions, the retention time of timicosin was 0.848 min, the recovery was 95%-103%, and the detection limit was 1ppb.

Table S1. Primers and PCR conditions for target genes.

Target genes	Primer（5′-3′）	Annealing temp (°C)	Amplicon size (bp)	Reference
16S rRNA	F: ACTCCTACGGGAGGCAGCGC<br>R: ATTACCGCGGCTGTGG	60	146	（Kumar et al., 2012）
*erm*A	F: GGTTTGCTATTGATGGTGGAA<br>R: GAACGCGATATTCACGGTTTA	60	154	(Kristiansson et al.,2011)
*erm*B	F: AAAACTTACCCGCCATACCA<br>R: TTTGGCGTGTTTCATTGCTT	55	185	（Kristiansson et al., 2011）
*erm*C	F: GAAATCGGCTCAGGAAAAGG<br>R: TAGCAAACCCGTATTCCACG	55	185	(Chen et al.,2007)
*erm*F	F: CGACACAGCTTTGGTTGAAC<br>R: GGACCTACCTCATAGACAAG	56	309	(Chen et al.,2007)
*erm*Q	F: CACCAACTGATATGTGGCTAG<br>R: CTAGGCATGGGATGGAAGTC	56	154	(Chen et al.,2007)
*erm*X	F: GCTCAGTGGTCCCCATGGT<br>R: ATCCCCCCGTCAACGTTT	61	488	
*mef*A	F: AGTATCATTAATCACTAGTGC<br>R: TTCTTCTGGTACTAAAAGTGG	60	348	
*tol*C	F: CGACACAGCTTTGGTTGAAC<br>R: GGACCTACCTCATAGACAAG	60	235	
*int*1	F: CCTCCCGCACGATGATC<br>R: TCCACGCATCGTCAGGC	55	280	(Like et al.,2019)
*int*2	F: GTTATTTTATTGCTGGGATTAGGC<br>R: TTTTACGCTGCTGTATGGTGC	57	164	(Kaiyu et al.,2019)
*tnp*A	F: CGCTTTGTTACGCCAGTC<br>R: TTCAGCACGCCATAGTCG	60	344	

*tn*916	F: GACAGTATTAAGCCATCAGAC<br>R: TCTTCCGAACACAATCATCT	50	146

Table S2. Mobile phase gradient elution procedure

time（min）	0.1% Formic acid water（%）	acetonitrile（%）
0	90	10
2	80	20
3	50	50
3.5	10	90
5	90	10
6	90	10

Table S3. Mass spectrum conditions for Timicosin

Antibiotic	Parent ion	Daughter ion	Collision voltage (V)	Collision energy (V)
Tilmicosin	869.6	696.4	280	45
		174.1	280	45

Table S4. Excretion rate of tilmicosin in the feces of pigs in each test group (%)

Group	CK	L	H
Total intake of tilmicosin（g）	0	27	54
Tilmicosin excretion during the dosing period（g/ head）	0	6.97±1.97	17.79±3.34
Total excretion of tilmicosin during the off-drug period（g/ head）	0	0.51±0.063	2.50±0.17
Total excretion of tilmicosin（g/ head）	0	7.48±1.68	20.29±2.79
Tilmicosin excretion rate during the dosing period（%）	0	25.82±3.31	32.93±7.72
Tilmicosin excretion rate during off-drug period（%）	0	1.99±0.05	4.63±0.08

Total excretion rate of tilmicosin（%）	0	27.82±2.46	37.56±4.58*

Table S5. Log values of ARGs and MGEs absolute abundance in pig manure from different days of treat groups (copies/g).

		0d		1d		6d		16d		18d		22d		29d		all samples	
Gene	group	mean± std. error	Detection rate/100%	mean± std. error	Detection rate/100%	mean± std. error	Detection rate/100%	mean± std. error	Detection rate/100%	mean± std. error	Detection rate/100%	mean± std. error	Detection rate/100%	mean± std. error	Detection rate/100%	mean± std. error	Detection rate/100%
	CK	8.41± 7.44	100	8.19± 7.31	100	8.1± 7.10	100	8.35± 7.32	100	8.44± 7.23	100	8.38± 7.02	100	7.74± 6.92	100	8.28± 6.43	100
*erm*A	L	8.09± 7.14	89	7.84± 7.25	100	8.38± 7.49	100	8.37± 7.22	100	8.04± 6.76	100	8.32± 7.34	100	7.7± 6.60	100	8.17± 6.43	98
	H	8.41± 7.44	100	8.19± 7.31	100	8.1± 7.10	100	8.35± 7.32	100	8.44± 7.23	100	8.38± 7.02	100	7.74± 6.92	100	8.28± 6.43	100
	CK	8.44± 7.76	89	7.61± 6.68	100	8.11± 7.15	100	8.77± 7.97	89	8.61± 7.45	100	8.55± 7.76	100	8.12± 7.24	100	8.44± 6.86	97
*erm*B	L	8.70± 8.16	89	7.66± 7.09	100	8.24± 7.38	100	8.21± 7.23	100	8.15± 7.03	100	8.32± 7.27	100	7.70± 6.63	100	8.26± 6.91	98
	H	7.34± 6.34	100	7.47± 6.56	100	8.46± 7.63	100	8.36± 7.38	100	8.33± 7.27	100	8.87± 8.33	100	7.54± 6.36	100	8.35± 7.08	100
	CK	7.70± 6.86	100	8.45± 7.89	100	8.41± 7.66	100	7.89± 7.00	100	8.08± 7.03	100	7.97± 7.01	100	7.98± 6.82	100	8.15± 6.69	100
*erm*C	L	7.90± 7.16	100	8.00± 7.40	100	8.81± 8.20	100	8.01± 7.11	100	7.96± 6.93	100	7.65± 6.82	100	7.69± 6.95	100	8.2± 6.95	100
	H	7.64± 6.80	100	7.72± 6.93	100	8.22± 7.47	100	7.74± 6.86	100	8.25± 7.50	100	7.54± 6.67	100	7.69± 6.86	100	7.92± 6.4	100

	CK	8.43± 7.14	100	7.80± 6.90	100	8.19± 6.98	89	8.31± 7.01	100	8.38± 7.22	100	8.17± 6.96	89	8.23± 7.14	100	8.25± 6.28	97
*erm*F	L	8.68± 7.63	100	7.80± 7.13	100	8.30± 7.30	100	8.11± 6.90	100	8.08± 6.81	100	8.40± 7.80	100	8.03± 7.07	100	8.28± 6.67	100
	H	8.56± 7.88	100	7.88± 7.21	100	8.30± 7.14	100	8.18± 6.93	100	8.27± 6.74	100	8.42± 7.24	100	8.22± 7.18	100	8.3± 6.65	100
	CK	8.53± 7.55	100	8.32± 7.58	100	8.15± 7.21	100	8.18± 7.33	100	8.26± 7.61	100	8.37± 7.56	100	7.86± 7.08	100	8.28± 6.63	100
*erm*Q	L	8.37± 7.49	100	7.36± 6.56	100	8.25± 7.50	100	8.10± 7.37	100	8.15± 7.36	100	7.94± 7.08	100	7.99± 7.15	100	8.10± 6.50	100
	H	8.03± 7.32	100	7.89± 7.14	100	8.07± 7.20	100	7.36± 6.59	100	7.84± 7.05	100	8.39± 7.53	100	7.89± 7.10	100	8.01± 6.43	100
	CK	7.45± 6.57	89	7.66± 7.07	100	7.74± 6.96	100	7.04± 5.99	100	7.30± 6.21	100	7.34± 6.31	100	7.29± 6.30	100	7.46± 5.93	98
*erm*X	L	7.00± 5.79	89	7.24± 6.62	100	7.94± 7.09	100	7.26± 6.28	100	7.57± 6.36	100	7.08± 5.85	100	7.22± 6.30	100	7.46± 5.91	98
	H	7.19± 6.28	100	7.52± 6.74	100	7.42± 6.41	100	7.14± 5.94	100	7.81± 6.98	100	7.13± 6.10	100	7.62± 6.60	100	7.47± 5.84	100
	CK	6.82± 5.70	100	6.12± 5.09	100	6.81± 5.87	100	7.13± 5.89	100	6.90± 5.89	100	7.04± 5.94	100	6.47± 5.55	100	6.85± 5.03	100
*mef*A	L	7.61± 6.60	100	5.95± 4.99	100	7.16± 6.17	100	6.84± 6.09	100	7.24± 6.17	100	6.79± 5.74	100	6.76± 6.03	100	7.12± 5.5	100
	H	7.00± 6.34	89	6.06± 5.28	89	6.93± 6.01	100	6.18± 5.41	100	6.88± 5.75	100	6.54± 5.20	100	6.23± 5.06	100	6.68± 5.13	97
	CK	8.45± 7.79	100	8.42± 7.87	100	8.78± 8.07	100	9.08± 8.05	100	9.63± 9.00	100	9.18± 8.63	100	9.33± 8.76	100	9.17± 7.83	100
*tol*C	L	9.23± 8.73	100	8.55± 8.05	100	8.09± 7.33	100	9.35± 8.70	100	9.73± 8.94	100	9.66± 9.10	100	9.25± 8.56	100	9.36± 7.97	100
	H	8.45± 7.82	100	8.38± 7.59	100	8.33± 7.72	100	9.42± 8.65	100	10.45± 9.74	100	8.77± 7.98	100	9.41± 8.64	100	9.69± 8.5	100
*int*1	CK	9.86± 8.87	100	10.14± 9.43	100	10.23± 9.30	100	10.54± 9.53	100	10.49± 9.33	100	10.27± 9.31	100	10.28± 9.50	100	10.31± 8.56	100

	L	9.86± 8.74	100	9.96± 9.38	100	10.26± 9.27	100	11.19± 10.66	100	10.78± 9.84	100	10.20± 9.47	100	10.48± 9.50	100	10.63± 9.4	100
	H	9.89± 8.97	100	10.11± 9.20	100	10.02± 9.18	100	10.99± 10.28	100	11.40± 10.53	100	10.31± 9.40	100	10.86± 10.14	100	10.83± 9.4	100
	CK	9.82± 8.97	100	10.04± 9.35	100	10.61± 9.97	100	10.20± 9.58	100	10.50± 9.95	100	9.76± 8.89	100	10.19± 9.43	100	10.26± 8.87	100
*int*2	L	10.37± 9.73	100	10.11± 9.55	100	10.61± 9.92	100	9.77± 8.82	100	10.29± 9.46	100	10.40± 9.78	100	10.10± 9.33	100	10.30± 8.83	100
	H	9.70± 8.89	100	10.04± 9.15	100	10.61± 9.81	100	10.18± 9.28	100	11.00± 10.25	100	10.18± 9.63	100	10.09± 9.28	100	10.46± 9.05	100
	CK	9.52± 8.84	100	9.57± 8.89	100	9.77± 9.08	100	10.55± 9.54	100	11.04± 10.49	100	10.52± 9.99	100	10.47± 9.87	100	10.50± 9.26	100
*tnp*A	L	9.17± 8.23	100	9.33± 8.80	100	9.49± 8.41	100	10.99± 10.33	100	10.64± 9.95	89	9.85± 8.99	89	10.24± 9.59	100	10.39± 9.12	97
	H	9.59± 8.83	100	9.45± 8.55	100	9.71± 8.97	100	10.75± 10.01	100	11.10± 10.20	100	10.01± 9.15	100	10.66± 9.97	100	10.55± 9.11	100
	CK	10.60± 9.79	100	10.61± 9.96	100	10.64± 9.75	100	10.12± 9.42	100	9.72± 9.03	100	10.14± 9.16	100	10.78± 10.04	100	10.49± 8.96	100
*tn*916	L	10.23± 9.48	100	10.69± 9.95	100	10.56± 9.80	100	10.46± 9.75	100	10.66± 9.84	100	10.17± 9.43	100	10.26± 9.37	100	10.48± 8.90	100
	H	10.23± 9.48	100	10.69± 9.95	100	10.56± 9.80	100	10.46± 9.75	100	10.66± 9.84	100	10.17± 9.43	100	10.26± 9.37	100	10.48± 8.90	100

Table S6. Log values of ARGs and MGEs relative abundance in pig manure from different days of treat groups (copies/g).

		0d		1d		6d		16d		18d		22d		29d		all samples	
Gene	group	mean± std. error	Detection rate/100%	mean± std. error	Detection rate/100%	mean± std. error	Detection rate/100%	mean± std. error	Detection rate/100%	mean± std. error	Detection rate/100%	mean± std. error	Detection rate/100%	mean± std. error	Detection rate/100%	mean± std. error	Detection rate/100%
	CK	-3.12± 4.04	100	-2.92± 3.52	100	-3.19± 4.14	100	-3.15± 4.18	100	-3.05± 4.21	100	-2.93± 4.13	100	-4.10± 5.16	100	-3.10±4.73	100
*erm*A	L	-2.84± 3.52	89	-2.80± 3.35	100	-2.93± 3.98	100	-3.05± 4.15	100	-3.43± 4.53	100	-3.05± 4.02	100	-3.88± 5.02	100	-3.03±4.52	98
	H	-2.85± 3.79	100	-3.34± 4.35	100	-3.38± 4.20	100	-3.20± 4.25	100	-3.02± 4.22	100	-2.93± 3.98	100	-3.83± 4.68	89	-3.13±4.88	98
*erm*B	CK	-3.28± 4.06	89	-2.83± 3.74	100	-3.33± 4.28	100	-2.76± 3.57	89	-2.89± 4.00	100	-2.77± 3.58	100	-3.47± 4.30	100	-2.96± 4.6	97

	L	-3.57± 4.44	89	-3.00± 3.50	100	-2.78± 3.59	100	-3.21± 4.15	100	-3.35± 4.37	100	-3.06± 4.02	100	-3.89± 5.00	100	-3.14± 4.64	98
	H	-3.94± 4.96	100	-3.37± 4.20	100	-2.89± 3.54	100	-3.10± 4.00	100	-3.09± 4.10	100	-2.54± 3.10	100	-4.05± 5.26	100	-3.04± 4.33	100
	CK	-4.05± 4.99	100	-2.55± 3.35	100	-3.07± 3.87	100	-3.60± 4.47	100	-3.38± 4.42	100	-3.33± 4.29	100	-2.54± 3.06	100	-2.96± 4.27	100
*erm*C	L	-3.34± 4.07	100	-2.67± 3.20	100	-2.69± 3.51	100	-3.42± 4.29	100	-3.53± 4.54	100	-3.87± 4.73	100	-3.87± 4.54	100	-3.10± 4.41	100
	H	-3.79± 4.67	100	-3.24± 3.95	100	-3.09± 3.68	100	-3.71± 4.56	100	-3.14± 3.84	100	-3.90± 4.78	100	-3.97± 4.87	100	-3.41± 4.82	100
	CK	-3.18± 4.44	100	-2.52± 3.26	100	-3.15± 4.02	89	-3.20± 4.54	100	-3.09± 4.16	100	-3.18± 4.45	89	-3.02± 3.70	100	-2.97± 4.48	97
*erm*F	L	-2.65± 3.59	100	-3.29± 4.12	100	-2.78± 3.80	100	-3.32± 4.47	100	-3.39± 4.62	100	-3.15± 3.77	100	-3.50± 4.36	100	-3.04± 4.66	100
	H	-2.87± 3.57	100	-2.81± 3.46	100	-3.32± 4.64	100	-3.31± 4.39	100	-3.17± 4.59	100	-2.98± 4.18	100	-3.37± 4.40	100	-3.07± 4.62	100

	CK	-3.12± 4.16	100	-3.33± 4.33	100	-2.99± 3.73	100	-3.38± 4.29	100	-3.44± 4.24	100	-2.98± 3.82	100	-3.09± 3.69	100	-3.16±4.75	100
*erm*Q	L	-2.77± 3.69	100	-3.43± 4.06	100	-2.80± 3.57	100	-3.30± 4.02	100	-3.24± 4.00	100	-3.43± 4.27	100	-3.60± 4.44	100	-3.12± 4.67	100
	H	-3.28± 4.07	100	-3.09± 3.90	100	-3.29± 3.93	100	-4.13± 4.85	100	-3.55± 4.30	100	-3.03± 3.89	100	-3.68± 4.47	100	-3.32± 4.88	100
	CK	-4.36± 5.26	89	-3.30± 4.18	100	-3.62± 4.36	100	-4.43± 5.44	100	-4.17± 5.24	100	-4.01± 5.05	100	-4.04± 4.92	100	-3.81± 5.32	98
*erm*X	L	-3.66± 4.46	89	-3.36± 3.96	100	-2.94± 3.62	100	-4.15± 5.09	100	-3.94± 5.14	100	-4.36± 5.56	100	-4.35± 5.26	100	-3.53± 4.83	98
	H	-4.12± 5.13	100	-3.63± 4.60	100	-4.08± 4.98	100	-4.37± 5.57	100	-3.57± 4.36	100	-4.31± 5.34	100	-3.99± 5.04	100	-3.91± 5.52	100
	CK	-4.78± 5.81	100	-4.23± 5.08	100	-4.62± 5.59	100	-4.37± 5.62	100	-4.62± 5.66	100	-4.31± 5.46	100	-4.03± 4.54	100	-4.36± 5.79	100
*mef*A	L	-3.18± 4.09	100	-5.03± 5.86	100	-3.90± 4.87	100	-4.57± 5.30	100	-4.21± 5.24	100	-4.58± 5.43	100	-4.82± 5.56	100	-3.88± 5.26	100

	H	-4.47± 5.12	89	-4.72± 5.41	89	-4.62± 5.53	100	-5.42± 6.33	100	-4.56± 5.66	100	-4.86± 6.19	100	-5.32± 6.31	100	-4.74± 6.30	97
	CK	-3.45± 4.28	100	-1.96± 2.47	100	-2.81± 3.55	100	-2.44± 3.45	100	-1.86± 2.50	100	-2.17± 2.72	100	-2.06± 2.72	100	-2.19± 3.55	100
*tol*C	L	-2.43± 3.08	100	-2.07± 2.55	100	-3.00± 3.74	100	-2.10± 2.77	100	-1.76± 2.50	100	-1.84± 2.40	100	-2.27± 2.95	100	-2.07± 3.48	100
	H	-2.50± 3.04	100	-2.97± 3.86	100	-2.89± 3.41	100	-2.13± 2.89	100	-1.37± 2.26	100	-2.63± 3.41	100	-2.18± 2.97	100	-2.06± 3.45	100
	CK	-1.83± 2.97	100	-0.62± 1.56	100	-1.13± 1.97	100	-0.97± 1.96	100	-0.98± 1.97	100	-1.07± 2.04	100	-1.58± 2.59	100	-1.03± 2.68	100
*int*1	L	-0.73± 1.58	100	-0.67± 1.22	100	-0.67± 1.43	100	-0.27± 0.82	100	-0.72± 1.67	100	-1.17± 1.83	100	-1.15± 2.14	100	-0.68± 2.04	100
	H	-1.20± 1.87	100	-0.95± 1.82	100	-1.31± 1.93	100	-0.55± 1.22	100	-0.12± 1.05	100	-1.12± 1.99	100	-0.76± 1.48	100	-0.66± 2.15	100
*int*2	CK	-1.91± 2.89	100	-0.74± 1.64	100	-0.71± 1.32	100	-1.33± 1.96	100	-1.00± 1.56	100	-1.57± 2.47	100	-1.07± 1.85	100	-1.03± 2.47	100

	L	-0.25± 0.89	100	-0.53± 1.04	100	-0.22± 0.79	100	-1.65± 2.56	100	-1.09± 1.85	100	-1.11± 1.72	100	-1.46± 2.22	100	-0.62± 1.91	100
	H	-1.37± 2.00	100	-1.21± 2.24	100	-0.91± 1.74	100	-1.30± 2.19	100	-0.47± 1.24	100	-1.42± 2.06	100	-1.57± 2.39	100	-1.01± 2.44	100
	CK	-2.37± 3.25	100	-0.89± 1.45	100	-1.77± 2.58	100	-0.96± 1.95	100	-0.46± 1.02	100	-0.84± 1.36	100	-0.92± 1.61	100	-0.90± 2.21	100
*tnp*A	L	-1.31± 2.01	100	-1.31± 1.80	100	-1.62± 2.65	100	-0.46± 1.13	100	-0.98± 1.80	89	-1.65± 2.53	89	-1.27± 1.90	100	-1.03± 2.35	97
	H	-1.47± 2.07	100	-1.74± 2.76	100	-1.57± 2.15	100	-0.80± 1.52	100	-0.40± 1.36	100	-1.40± 2.27	100	-0.96± 1.69	100	-0.95± 2.44	100
	CK	-1.15± 2.21	100	-0.30± 1.22)	100	-0.75± 1.60	100	-1.38± 2.12	100	-1.75± 2.47	100	-1.15± 2.11	100	-0.72± 1.47	100	-0.82± 2.34	100
*tn*916	L	-0.49± 1.18	100	-0.86± 1.64	100	-0.29± 0.88	100	-0.97± 1.64	100	-0.90± 1.91	100	-1.21± 1.97	100	-1.30± 2.11	100	-0.73± 2.08	100
	H	-1.09± 1.96	100	-0.53± 1.42	100	-0.91± 1.68	100	-1.02± 1.71	100	-0.80± 1.61	100	-1.33± 2.17	100	-1.36± 2.23	100	-0.92± 2.51	100

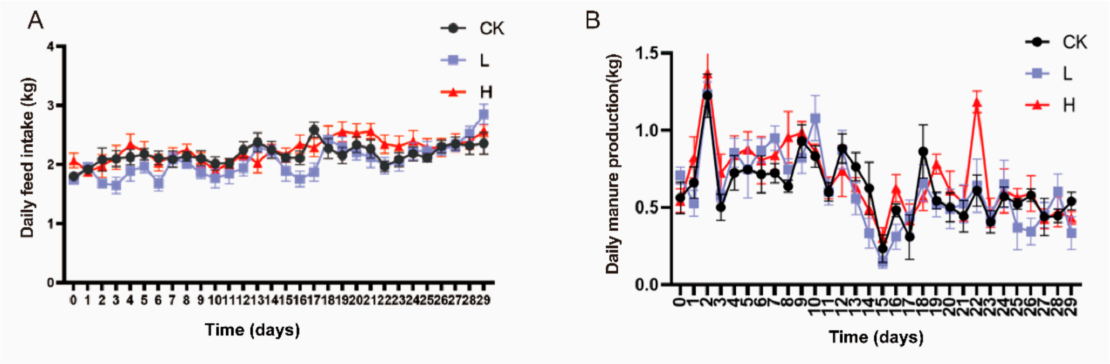

Figure S1. Effect of tilmicosin on daily feed intake (A) and daily manure production in pigs (B).

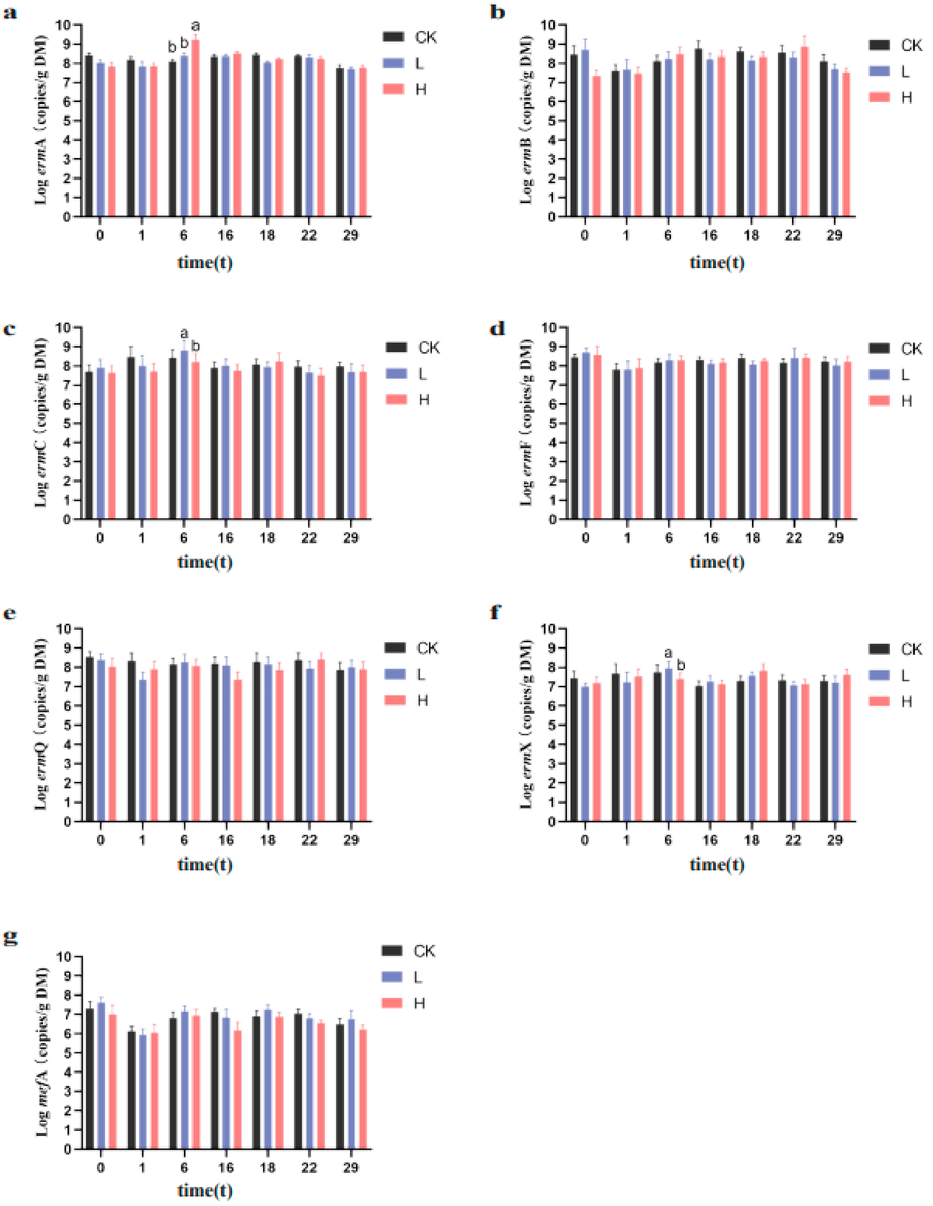

Figure S2. Absolute abundance of MRGs in pig manure from different days of treatment groups.(a) Absolute abundance of *erm*A; (b) Absolute abundance of *erm*B; (c) Absolute abundance of *erm*C; (d) Absolute abundance of *erm*F; (e) Absolute abundance of *erm*Q; (f) Absolute abundance of *erm*X; (g) Absolute abundance of *mef*A.

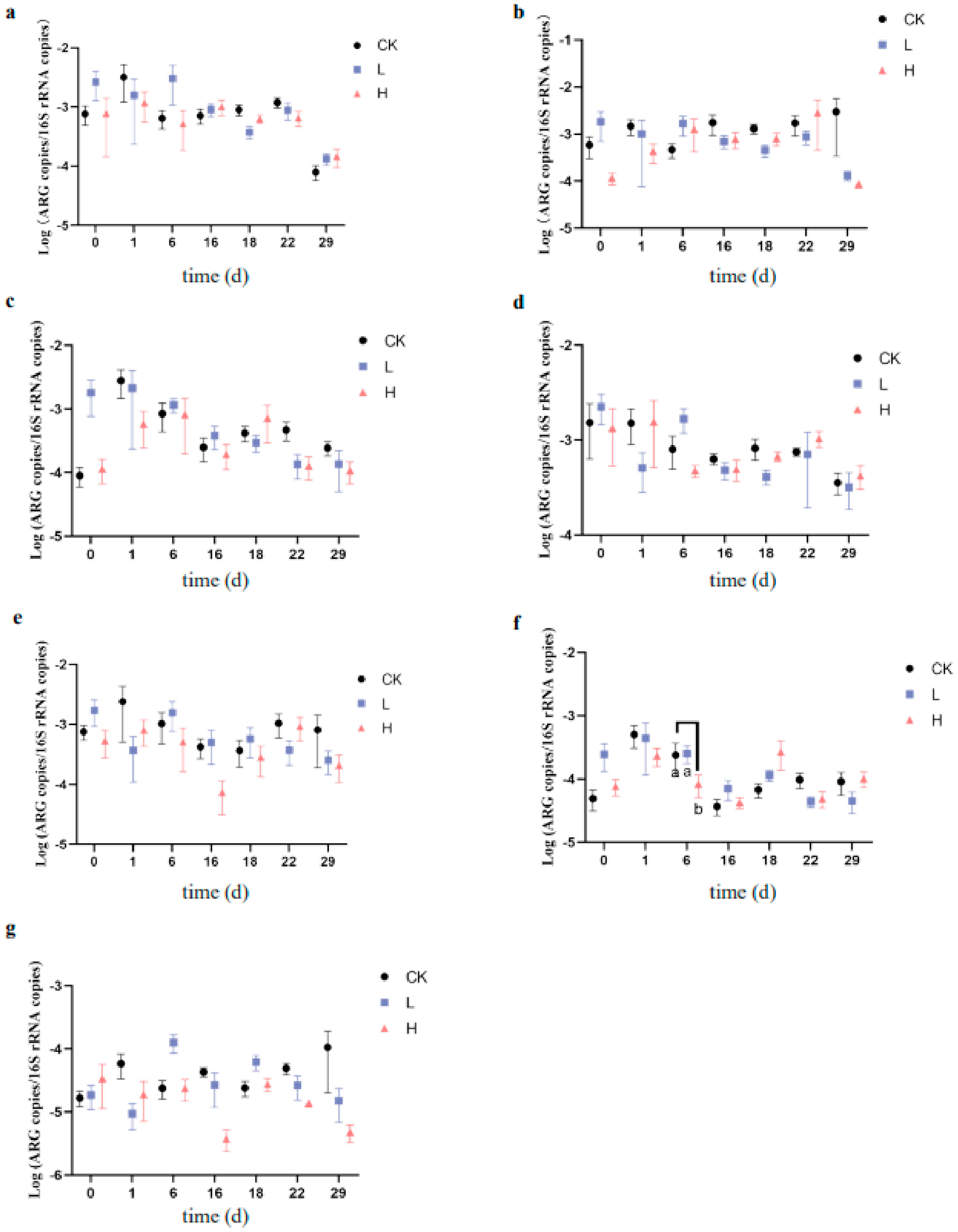

Figure S3. Relative abundance of MRGs in pig manure from different days of treatment groups. (a) Relative abundance of *erm*A; (b) Relative abundance of *erm*B; (c) Relative abundance of *erm*C; (d) Relative abundance of *erm*F; (e) Relative abundance of *erm*Q; (f) Relative abundance of *erm*X; (g) Relative abundance of *mef*A.

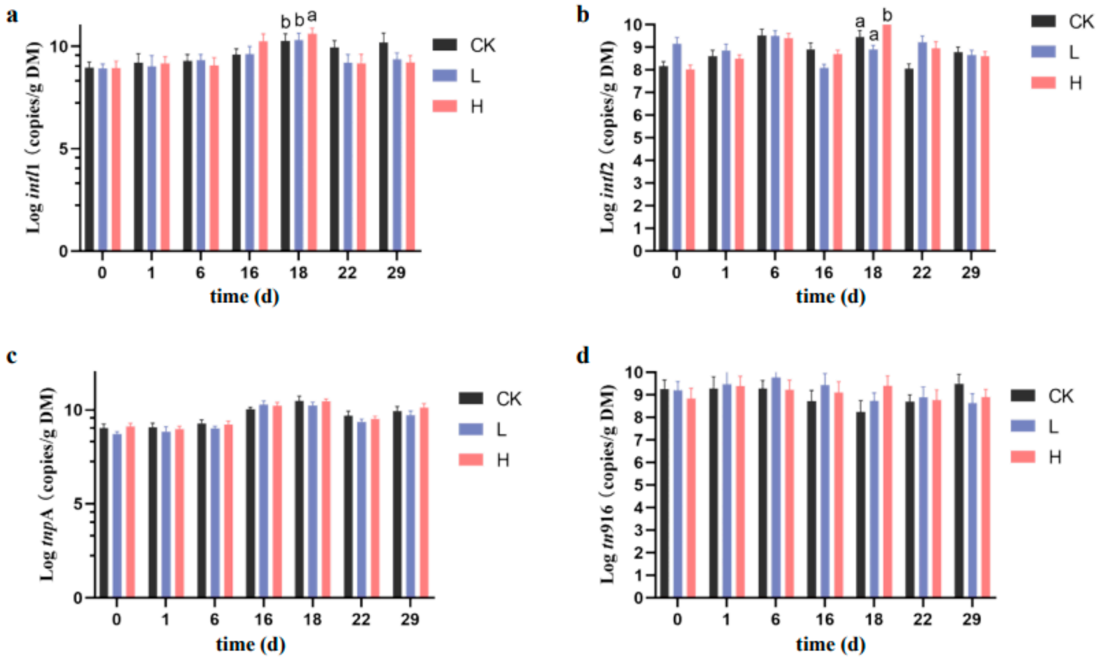

Figure S4. Absolute abundance of MGEs in pig manure from different days of treatment groups. (a) Absolute abundance of *int*1; (b) Absolute abundance of *int*2; (c) Absolute abundance of *tnp*A; (d) Absolute abundance of *tn*916.

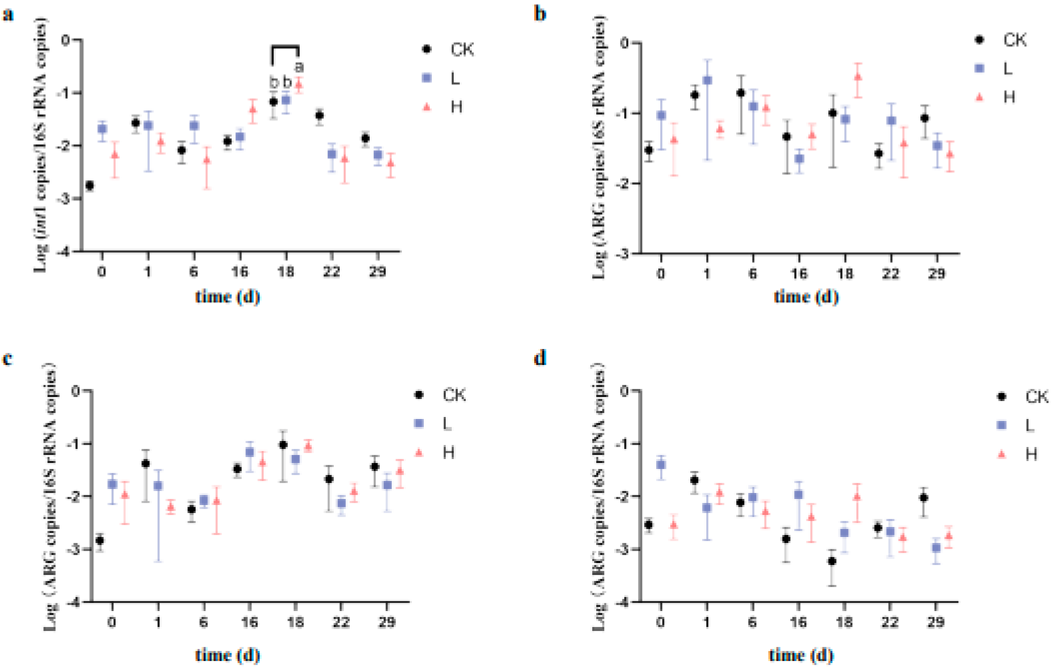

Figure S5. Relative abundance of MGEs in pig manure from different days of

treatment groups. (a) Relative abundance of *int*1; (b) Relative abundance of *int*2; (c) Relative abundance of *tnp*A; (d) Relative abundance of *tn*916.

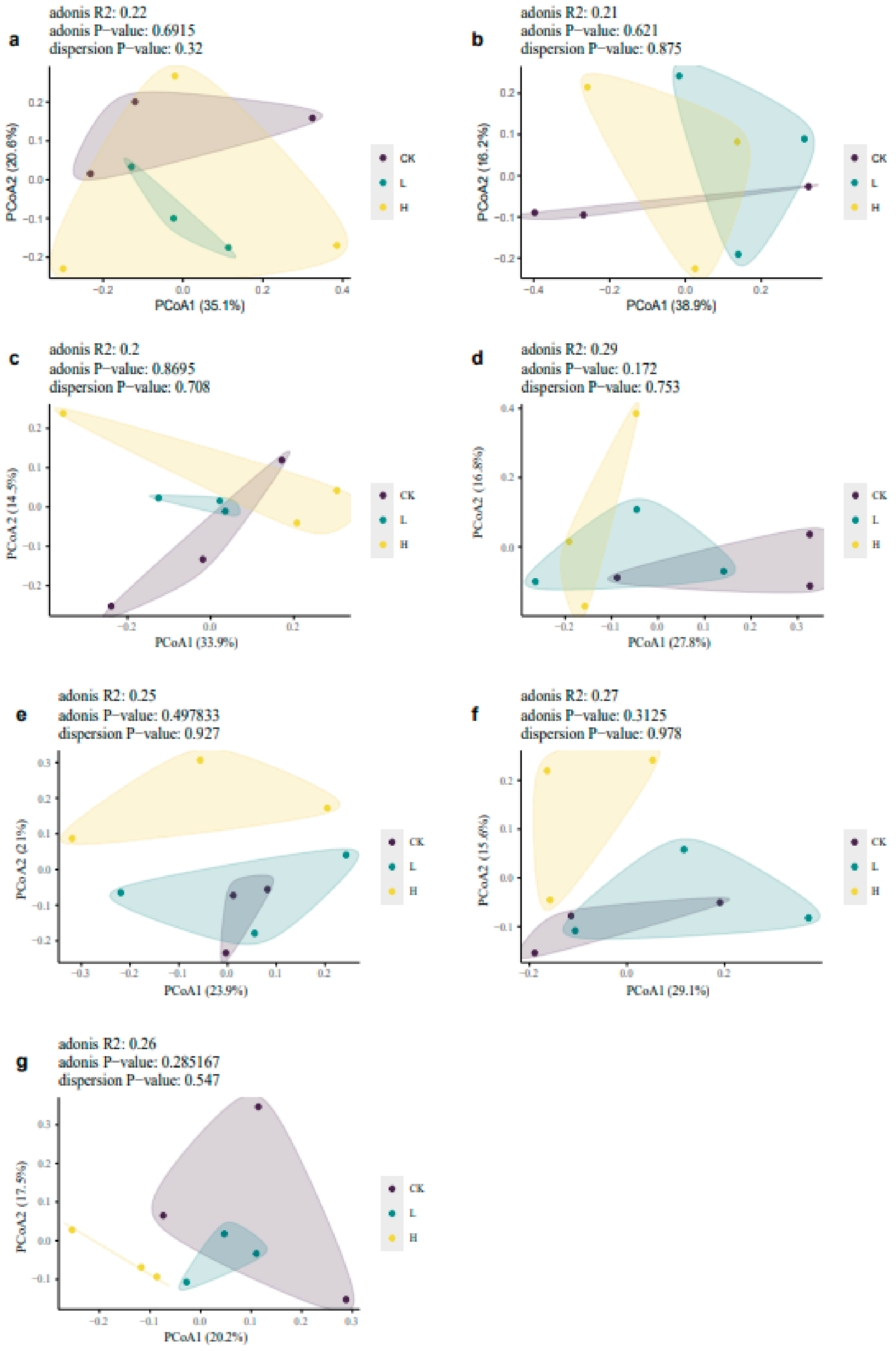

Figure S6. Analysis of β-diversity of pig manure in each experimental group. (a) day 0; (b) day 1; (c) day 6; (d) day 16; (e) day 18; (f) day 22; (g) day 29.

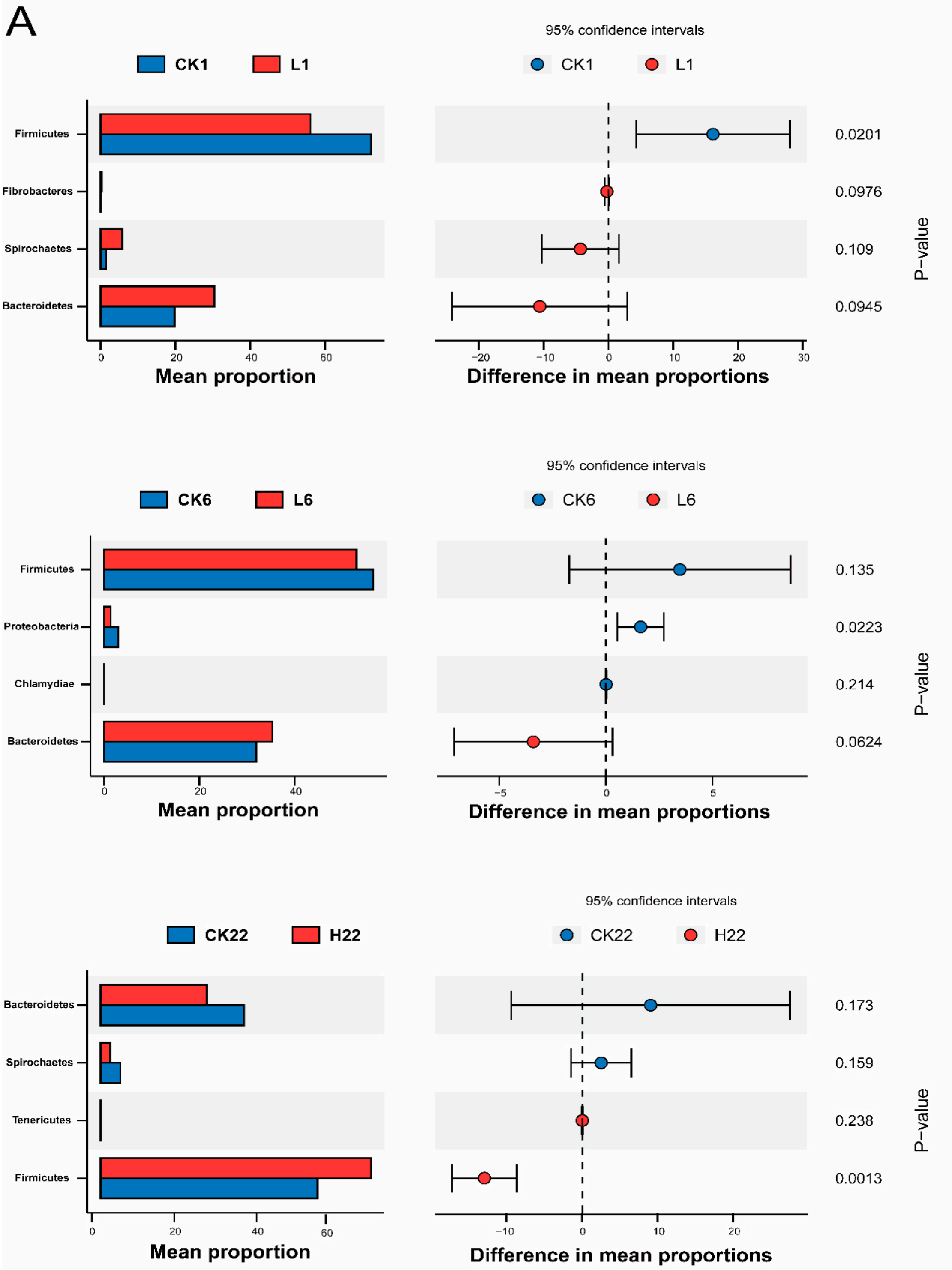
A
95% confidence intervals
CK1
L1
Firmicutes
Fibrobacteres
Spirochaetes
Bacteroidetes
Mean proportion
Difference in mean proportions
0.0201
0.0976
0.109
0.0945
P−value
95% confidence intervals
CK6
L6
Firmicutes
Proteobacteria
Chlamydiae
Bacteroidetes
Mean proportion
Difference in mean proportions
0.135
0.0223
0.214
0.0624
P−value
95% confidence intervals
CK22
H22
Bacteroidetes
Spirochaetes
Tenericutes
Firmicutes
Mean proportion
Difference in mean proportions
0.173
0.159
0.238
0.0013
P−value

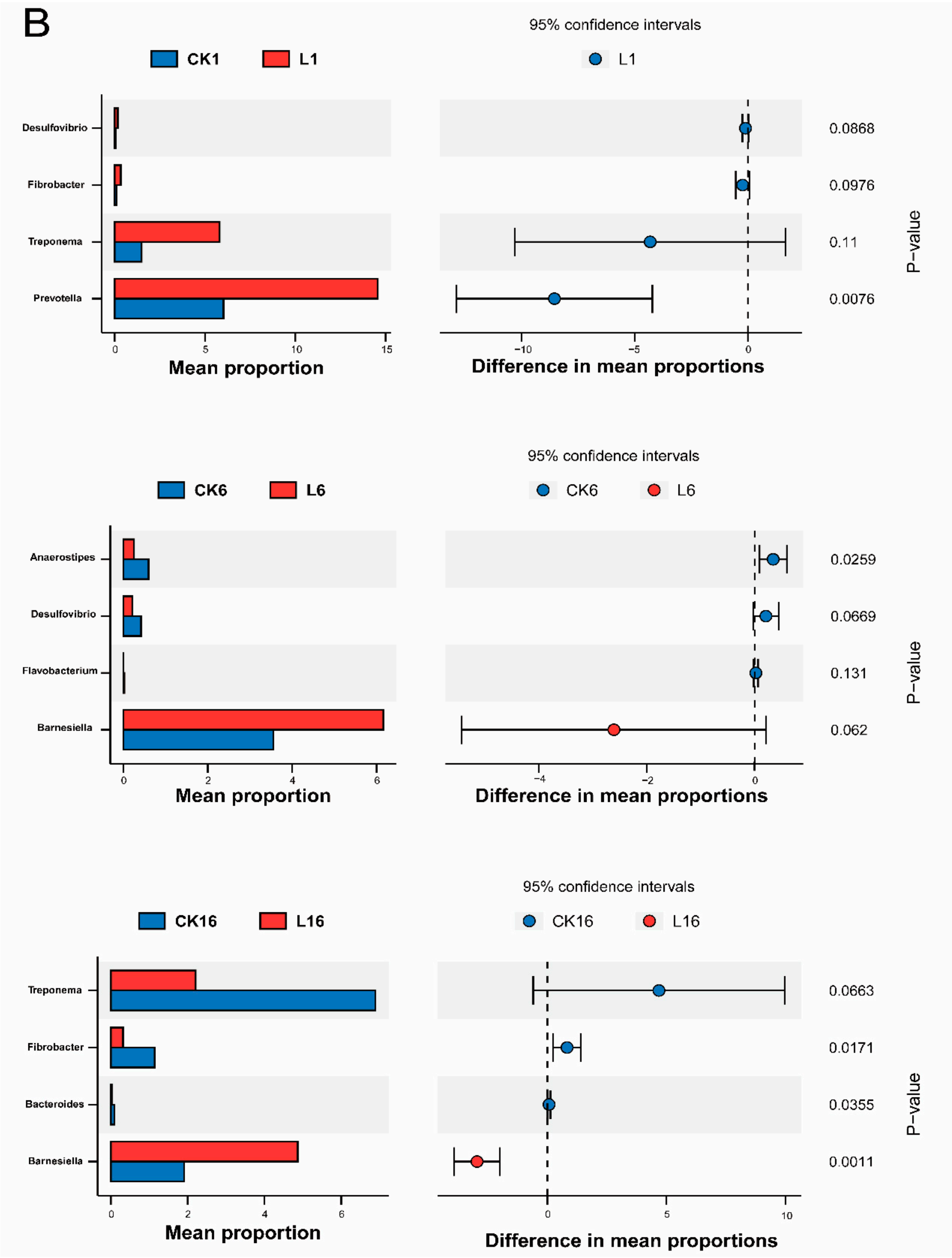
B
95% confidence intervals
CK1
L1
L1
Desulfovibrio
Fibrobacter
Treponema
Prevotella
0.0868
0.0976
0.11
0.0076
P−value
Mean proportion
Difference in mean proportions
95% confidence intervals
CK6
L6
CK6
L6
Anaerostipes
Desulfovibrio
Flavobacterium
Barnesiella
0.0259
0.0669
0.131
0.062
P−value
Mean proportion
Difference in mean proportions
95% confidence intervals
CK16
L16
CK16
L16
Treponema
Fibrobacter
Bacteroides
Barnesiella
0.0663
0.0171
0.0355
0.0011
P−value
Mean proportion
Difference in mean proportions

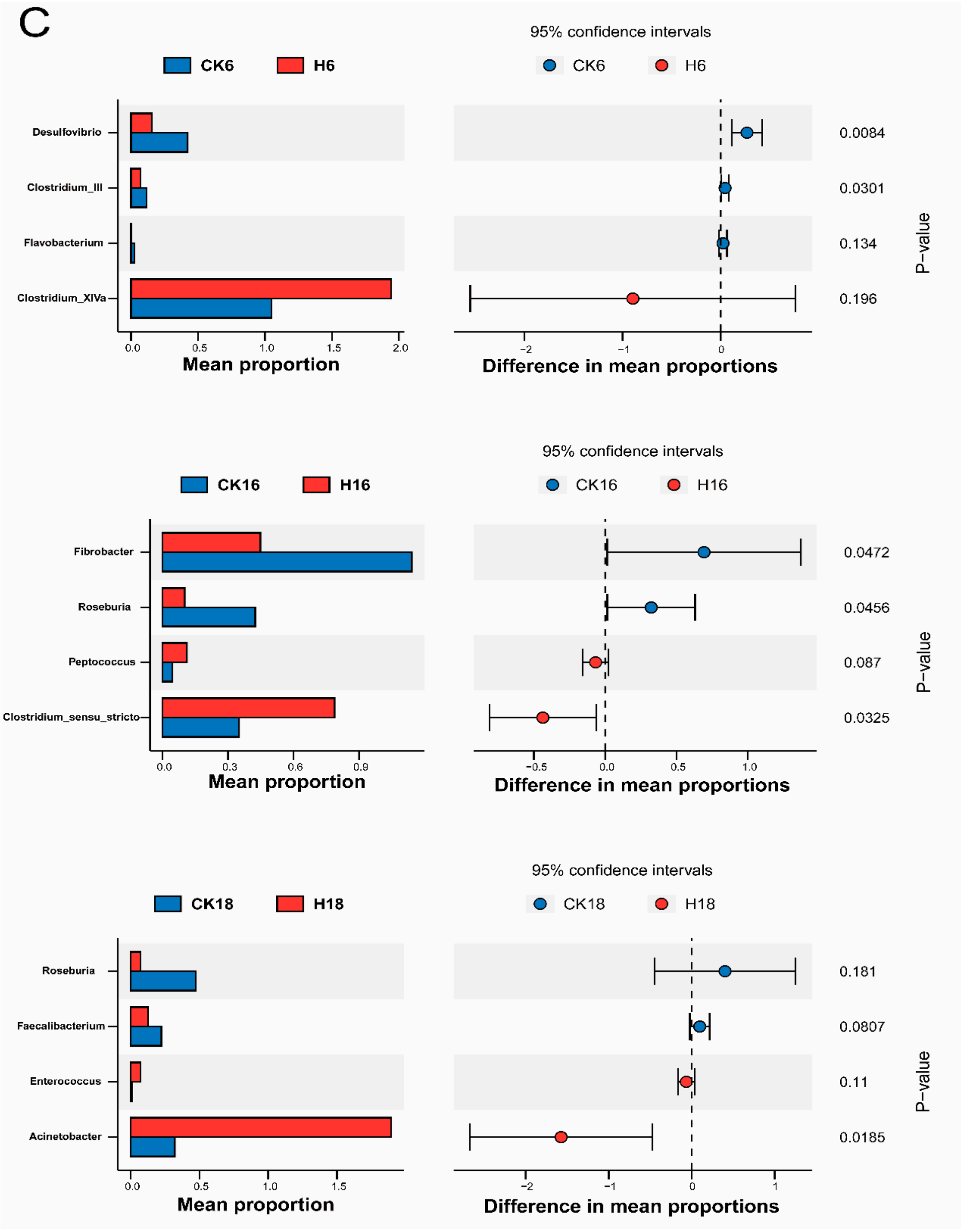

Figure S7. The difference between the three groups at the phylum and genus level. (A) Differences at the phylum level between L, H and CK groups; (B) Differences in genus level between L and CK groups; (C) Differences in genus level between H and CK groups

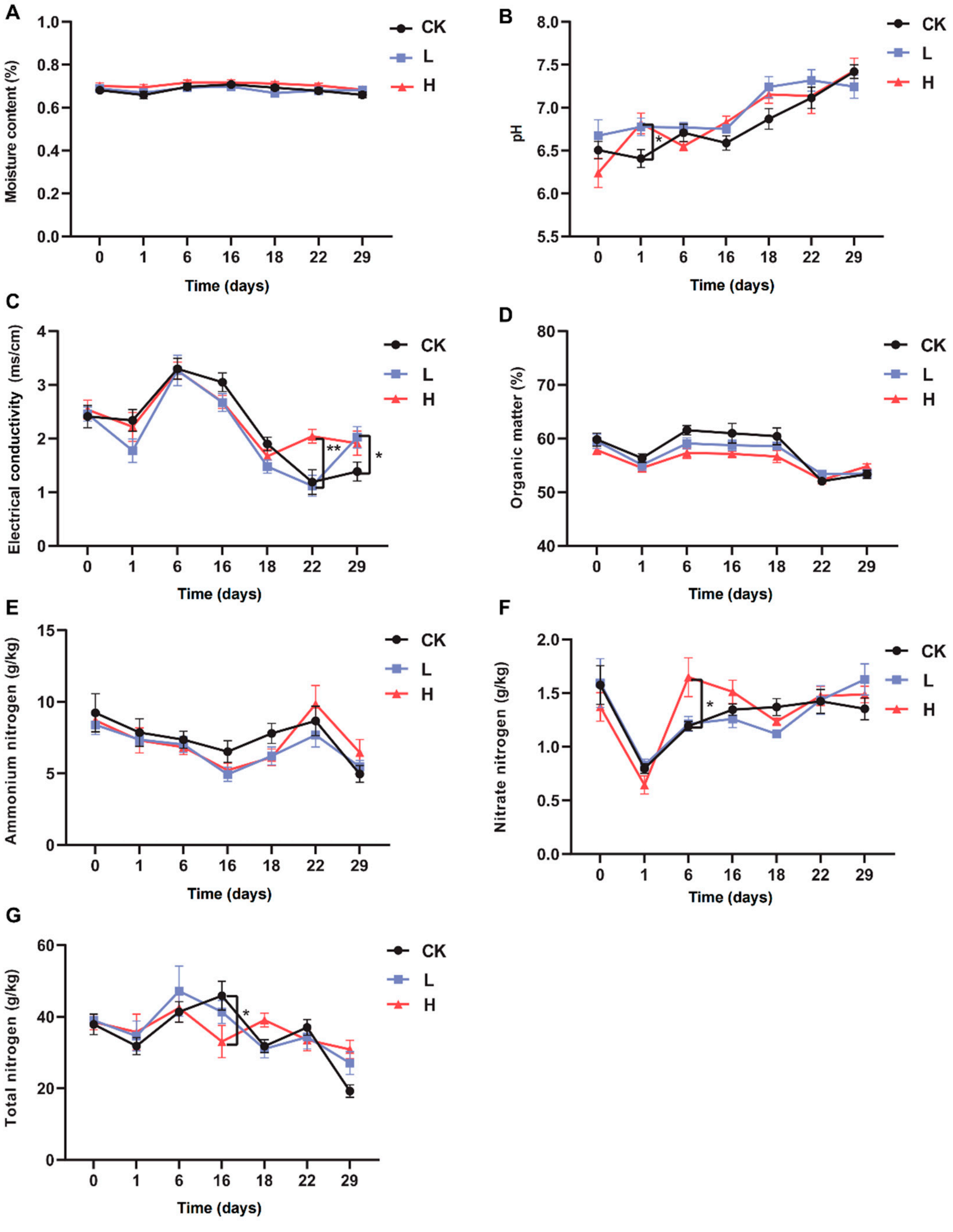

Figure S8. Changes in the main physicochemical properties of pig manure during the experiment. (A) MC; (B) pH; (C) EC; Carbon-nitrogen ratio (C/N); (D) OM; (E) NH_4^+-N; (F) NO_3^--N; (G) TN. *: 0.01 < P < 0.05, **: P < 0.01, ***: P < 0.001.
